# Evidence and gap maps: a comparison of different approaches

**DOI:** 10.4073/cmdp.2018.2

**Published:** 2018-10-12

**Authors:** Ashrita Saran, Howard White

## INTRODUCTION

Evidence maps are a relatively new approach to systematically identify and report the range of research activity in broad topic areas or policy domains. Although they go under various names, they have the common feature of adopting a systematic approach to mapping begins with a broad question, theme or issue which defines the scope of the evidence map. For reasons discussed below, we adopt the term Evidence and Gap Map to refer to the specific approach being proposed for Campbell, and evidence map to refer collectively to the range of approaches which have been adopted. As we describe in Part 2, evidence mapping of various forms has been around for at least 15 years.

A range of different approaches to evidence mapping and synthesis have been developed to support evidence informed policy making. In 2016, (Miake‐Lye, Hempel, Shanman, & Shekelle, 2016) conducted a systematic review of evidence maps in the health sector. The authors identified 145 titles, of these, 34 publications explicitly presented evidence maps and five publications used a mapping methodology without presenting a map. The authors conclude that there is diversity in definition and methods for mapping, so that stakeholders cannot necessarily know what to expect if they commission an evidence map or seek to identify existing maps. In addition, as there is no repository for evidence maps, maps are difficult to locate and authors are less likely to build on existing approaches. One purpose of this paper is to propose some general principles for evidence maps.

Maps often provide a foundation for further, more focused research synthesis. They are used to guide users to high quality research, inform research priority setting, and help to define the focus of evidence synthesis such as systematic reviews (Katz, et al., 2003). In the health sector maps were intended to provide clinicians, researchers, clients, and caregivers with tools and guidance to engage in evidence‐based decision making. Maps allow for identification of research gaps which is particularly important for interventions that are implemented without insufficient evidence (Katz, et al., 2003).

The popularity of mapping has grown in recent years. A map of maps for international development interventions identified 73 maps, with half of all completed maps commissioned in 2016 (Phillips, et al., 2017).

This paper reviews the range of mapping methodologies being adopted across different sectors. The motivation for this paper is to develop general principles for maps, and particularly to inform the development of standards for the Campbell Collaboration as a basis for the publication of Evidence and Gap Maps in the Campbell Library. The approaches discussed include systematic map (Bates, Clapton, & Coren, 2007); (Oakley, Gough, Oliver, & Thomas, 2005); (Salina Bates, 2006); (Peersman, 1996) evidence maps (Liu, Parker, Hetrick, & Callahan, 2010), the evidence‐based policing matrix (Lum, Koper, & Telep, 2011), Evidence Mapping (Parkhill, et al., 2011); (Bragge, et al., 2011); Scientific uncertainties (Jacobson, 2014), and Evidence Gap Maps (Snilstveit, Vojtkova, Bhavsar, & Gaarder, 2013).

Papers mentioned above have discussed and presented overview of different types of evidence mapping, scoping and synthesis. The focus is on the various approaches towards evidence syntheses products whereas the focus of this paper is on the methodological approach of organisations working on evidence maps.

The purposes of this paper are to:

- Identify various organisations that produce evidence maps
- Study the different approaches used for generating the evidence maps.
- Identify uniqueness and similarities in each step (Inclusion criteria, screening, search, coding and use)
- List all the ways in which evidence map can be generated
- Discuss areas of commonality and dissimilarity for each step between the different organizations.
- Propose some common standards for evidence maps.

We present a brief history of evidence mapping before turning to an analysis of the approach to evidence mapping broken down into stages: definition of map, scope, inclusion criteria, critical appraisal, screening and searching, coding, presentation and reporting, use and maintenance. We conclude with some general principles for evidence mapping.

## BACKGROUND

### Brief history of evidence mapping

The earliest evidence maps we identified are from 2003. That year saw publication of a map by the Yale Prevention Research Centre in collaboration with Complementary and Alternative Medicine (CAM) (Katz, Williams, Girard, & Goodman, 2003); see Table 1. The same year the Evidence for Policy and Practice Information and Coordinating Centre (EPPI‐Center) published a review of personal development planning for improving student learning, distinguishing between the systematic map of the research which been undertaken and the systematic synthesis of what the evidence says (Gough, Kiwan, Sutcliffe, Simpson, & Houghton, 2003). Two years later the first visual map was published as the Evidence Based Policing Matrix (Rosenberg & Knox, 2005).

**Table 1:** Table showing history of evidence since the inception of term evidence mapping

**Year**	**Organisation**	**Brief on inception of evidence mapping**
**2003**	Yale Prevention Research	Yale Prevention Research Centre in collaboration with Complementary and Alternative Medicine (CAM) developed a systematic and replicable 9‐step process termed “evidence mapping”(Katz, et al., 2003).
** **	Evidence for Policy and Practice Information and Co‐ordinating Centre (EPPI‐Centre)	EPPI‐Centre publish map of the effectiveness of personal development planning for improving student learning (Gough, Kiwan, Sutcliffe, Simpson, & Houghton, 2003).
**2005**	Evidence Based Policing Matrix (EBPM)	The Matrix originally emerged from work by Lum and Koper, who initially conceptualized it to discuss how crime prevention might be applied to counterterrorism. Inspired by Rosenberg and Knox's (Rosenberg & Knox, 2005) three‐dimensional grid for conceptualizing childhood well‐being and youth violence prevention, they created a Crime Prevention Matrix to map evaluated criminal justice interventions according to their common strategic and tactical characteristics (Lum, Koper, & Telep, 2011).
**2005**	Evidence for Policy and Practice Information and Co‐ordinating Centre (EPPI‐Centre)	Methodology for systematic mapping developed by EPPI (Oakley, Gough, Oliver, & Thomas, 2005)
**2006**	The Social Care Institute for Excellence (SCIE)	The EPPI‐Centre mapping methodology adapted by the Social Care Institute for Excellence (SCIE) in response to a lack of empirical data to answer specific questions using systematic review methodology, and a need for methodology to describe the literature in a broad field of interest (Salina Bates, 2006).
**2010**	International Initiative for Impact Evaluation (3IE)	The first 3ie “Evidence Gap Map” was produced which focused on the health and nutritional impact of agricultural interventions (Gaarder, 2010).
**2011**	National Trauma Research Institute‐ Global Evidence Mapping Initiative (GEMI)	The GEM initiative (established in 2007) is a collaborative effort involving the Australasian Cochrane Centre, the National Institute of Clinical Studies and National Institute of Communication Technology Australia, the University of Melbourne, Southern Health and Melbourne Health to provide an overview on existing research about Traumatic Brain Injury (TBI) and Spinal Cord Injury (SCI).(Bragge, et al., 2011)
**2012**	Collaboration for Environmental Evidence (CEE)	Evidence mapping adopted by Centre for Collaboration of Environmental Science.(Randall & James, 2012) It catalogued literature relating to the impacts of integrated farm management, organic farming and agri‐environment schemes on biodiversity in temperate ecosystems.
**2012**	Epistemonikos Foundation	Epistemonikos was founded by Gabriel Rada and Daniel Pérez in 2009 Pontificia Universidad Católica de Chile. Epistemonikos was officially launched in Spanish on April 20th, 2012 and internationally on August 14th. Epistemonikos has over 250 active collaborators that continuously upload and translate documents (Rada, Pérez, & Capurro, 2012)
**2014**	IZA Institute of study of Labour Economics, World of Labour (IZA)	World of Labour publishes literature reviews accompanied by maps and was launched on 1^st^ May 2014. (IZA World of Labor Launch) (Cameron L., 2014)
**2014**	Department of veteran affairs (Health services research and development)	VA's Health Services Research and Development (HSR&D) service completed four evidence maps relating to complementary and alternative medicine or integrative medicine modalities: mindfulness, tai chi, yoga, and acupuncture in the year 2014.(Office of Research & Development, 2015)
**2016**	Sightsavers	The visual impairment evidence gap maps (EGMs) have been developed by a team of researchers from Sightsavers, the Cochrane Eyes and Vision Group and the International Centre for Eye Health at the London School of Hygiene and Tropical Medicine, drawing on the guidance and previous experiences of the International Initiative for Impact Evaluation (3ie), which initiated the development of EGMs.(Virendrakumar, Jolley, Gordon, Bascaran, & Schmidt, 2016)
**2016**	International Rescue Committee (IRC)	The Evidence Maps were developed using funding from the UK Department for International Development, and are based on a format adapted from the International Initiative for Impact Evaluation's (IRC, 2016)
**2017**	Campbell Collaboration	Campbell pilots Evidence and Gap maps, publishing them in the Campbell Library.

In 2005 EPPI‐Centre published a mapping methodology which was adapted the following year by the Social Care Institute for Excellence (SCIE) in response to a lack of empirical data to answer specific questions using systematic reviews, and a perceived need for a methodology to describe the literature in a broader field of interest than that typically covered in a single review.

In 2010, 3ie produced its first evidence map, labelled the Evidence Gap Map. The organisation's experience in preparing maps for a range of topics in international development is reviewed by Snilstveit (Snilstveit, Vojtkova, Bhavsar, & Gaarder, 2013). Several organizations in the international development sphere have since worked with 3ie, or independently, to develop their own evidence maps which typically have variations in design. These include International Rescue Committee (IRC, 2016), USAID (Vinogradova & Heaner, 2016), Sightsavers (Virendrakumar, Jolley, Gordon, Bascaran, & Schmidt, 2016), BEAM Exchange on Market Research.

Each of the agencies producing maps has a different approach, and each defines evidence map differently. A review of the definitions (see Annex 1 for full definitions) shows the following eight components contained in the definitions (see Table 2)^1^:

**Table 2:** Components of evidence map definitions

** **	**Systematic**	**Type of evidence**	**Content**	**Structure**	**Transparent**	**Visual display**	**Descriptive report**	**Users**
**EBPM**		X	X	X		X		X
**EPPI**			X	X	X		X	
**SCIE**				X	X		X	
**3ie**		X	X	X		X		
**GEMI**	X		X			X		
**CEE**	X		X	X	X		X	
**SBU**	X		X		X	X	X	X
**Department of Veteran affairs**	X	X	X	X		X		X
**Epistemonikos**		X	X	X		X		
**IZA World of Labour**		X				X		
**IRC**		X	X	X		X		
**Beam Exchange**		X	X	X				
**Sightsavers**		X	X	X		X		X
**Overseas Development Institute (ODI)**			X	X		X	X	
**UNICEF Innocenti**		X	X	X	X	X

- Systematic
- The type of evidence included
- The content of the map
- The structure of the map
- Transparency
- Visual or graphical display
- Accompanying description of map
- Intended users

What is perhaps missing in the definitions is a more explicit statement that mapping commonly tells us what evidence there is, but not what this evidence says ‐ though some maps do include information on effects. This point is often misunderstood by those commissioning evidence maps and so should be made clear up front where it is the case.

From these definitions we suggest the following comprehensive definition. Elements in square brackets […] may be considered optional.

*‘An evidence and gap map is a systematic [visual] presentation of the availability of relevant evidence [of effects] for a particular policy domain. The evidence is identified by a search following a pre‐specified, published search protocol. [The map may be accompanied by a descriptive report to summarize the evidence for stakeholders such as researchers, research commissioners, policy makers, and practitioners] [Evidence maps summarize what evidence there is, not what the evidence says]’.*

### Locating evidence and gap maps in the evidence synthesis ecosystem

Evidence and gap maps have two sets of relatives. The first relatives are other evidence synthesis research outputs. The second are different evidence platforms.

**Figure 1:**
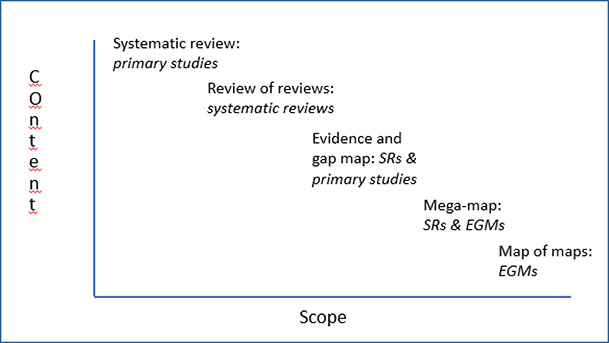
Scope and content of different evidence synthesis products

Figure 1 shows different evidence synthesis products, and the inverse relationship between scope and content. Systematic reviews are narrowest in scope but deep on content, coding, analyzing and reporting effect sizes, intervention characteristics and contextual factors. A review of reviews may be broader in scope but may be more restricted in the depth of analysis. Evidence and gap maps have a still broader scope but report a far more limited range of information about the reviews and primary studies they include. A mega‐map is a map with a very broad scope. It shows other maps and reviews, but not primary studies. And a map of maps only reports other EGMs in that policy space (Phillips, et al., 2017). Evidence maps are a ‘break’ on the content spectrum, as reviews and reviews of reviews report what the evidence says whereas many maps report what evidence there is but not what it says.

Evidence maps may be a way of curating (or brokering) a body of evidence to make it accessible to users. We distinguish three main evidence platforms with successively higher levels of curation or brokering:

- Evidence databases which contain evidence relating to a specific sector, e.g. ERIC for education, Epistimonikos for health, and the 3ie database for international development. Evidence databases differ from library catalogues and general databases, such as Scholar Google, since they are oriented to a particular audience, and possibly particular types of evidence. Both Epistimonikos and the 3ie database are restricted to systematic reviews and primary studies of effects.
- Evidence maps which classify the evidence which relates to a particular sector or issue, including some reporting of the features (but not content) of the evidence.
- Evidence portals which present evidence findings in a way in which is intended to be accessible to policy makers and practitioners. Examples are the Teacher and Learning Toolkit of the Education Endowment Foundation, and the IES's What Works Clearing House.

Beyond evidence portals there are evidence‐based guidelines and checklists which contain recommendations and directions. These last three are different from databases and maps as databases and maps take users back to the research studies, whereas portals etc. present the evidence to inform decision‐making without the user needing to consult the research directly.

This paper is focused on evidence maps. A companion paper will discuss evidence portals.

### Scope of EGMs

For nearly all the agencies reviewed, evidence maps generally have a broad thematic scope covering a range of interventions and outcomes. For example, 3ie maps generally cover a sector or broad sub‐sector such as forest conservation (Puri, Nath, Bhatia, & Glew, 2016), youth and transferable skills (Rankin, et al., 2015), peacebuilding (Cameron, et al., 2015), and primary and secondary education. SBU has mapped gaps (‘uncertainties’) for topics such as attention deficit hyperactivity disorder (ADHD), home care, and evidence‐based nursing for treatment of people with schizophrenia.

Department of Veteran Affairs has topics such as ‘Women veteran health research literature’(Elisheva, et al., 2017) and ‘Evidence map on mindfulness’ (Hempel, et al., 2014). Since 2007, the Global Evidence Mapping Initiative has mapped 1,644 neurotrauma publications, covering fifty‐three high priority questions (Bragge, et al., 2011). An example of evidence map by Global Evidence Mapping Initiative is ‘Social and communication disorder following brain injury’. Social Care Institute for Excellence maps cover areas such as the extent and impact of parental mental health problems on families, and the acceptability Salina Bates, 2006), accessibility and effectiveness of interventions, second examines the recovery approach in day services in adult mental health care (Sharif, Brown, & Rutter, 2008).

As of January 2018, the Epistemonikos database contains over 115,000 documents, of which 22,649 are systematic reviews, 76,834 are primary studies and 17,144 are structured summaries of evidence. The database is available in nine different languages: Arabic, German, English, Spanish, Italian, Dutch, Portuguese, Chinese, and French. It contains 7,266 official translations, 642 collaborative translations and 899,022 automated translations (Rada, Pérez, & Capurro, 2012). The database supports maps of the evidence found by database searches – these maps show which primary studies are reported in which systematic reviews found from a particular search.

IZA's World of Labour presents reviews on issues related to labour market policy and related economic issues such as ‘Do immigrant workers depress the wages of native workers?’(Giovanni, 2014). The reviews include an actual geographic map of where the evidence comes from.

The scope of Sightsaver's evidence map is limited to eye health. They have published five evidence maps so far, on cataract, diabetic retinopathy, glaucoma, trachoma and refractive error. The Beam Exchange evidence map is focused on market‐based approaches to promote economic development, improve access to services and reduce poverty (Robinson & Rust‐Smith, 2017).

The IRC has published five evidence maps covering the organization's five main outcomes areas: health, education, economic wellbeing, safety, and power. In addition, there are three cross‐cutting maps on cash transfer interventions, service delivery interventions, and interventions in humanitarian emergencies.

The scope of the evidence maps of the Collaboration for Environmental Evidence is typically broader. Examples include woodfuel value chain (Sola, et al., 2017) and farm land abandonment at high altitude (Haddaway, Styles, & Pullin, 2014).

An exception to this general broad scope of maps is the Evidence Based Policing Matrix which has just one outcome (reducing crime and disorder) rather than a set of outcomes.

ODI (Marcus & Cunningham, 2016) summarized evidence concerning initiatives and outcomes in four evidence maps: one providing a visual overview of all the evidence in the database, one focused on initiatives with young people acting as agents, one on young people as advocates, and one summarising evidence on initiatives that involved both agents and advocates activity.

UNICEF‐Innocenti have published an evidence gap map (EGM) of the evidence base for adolescent interventions in low‐ and middle‐income countries (LMICs). The thematic scope broadly corresponds with the UNICEF adolescent well‐being outcome domains of protection, participation and livelihoods.

### Inclusion criteria

All the agencies reviewed state pre‐specified inclusion criteria for their evidence maps. Inclusion criteria for maps use the PICOS approach of population, intervention, relevant comparison groups (effectiveness studies only, though specifying comparisons is usually not included in map inclusion criteria), outcomes, and eligible study designs. In practice, there will usually be some iteration between the studies identified and finalizing the intervention and outcome categories.

Most agencies produce maps including both systematic reviews and primary studies. Exceptions are the Evidence‐Based Policing Matrix which only includes primary studies, and both SBU and Sightsavers maps include only systematic reviews.

3ie EGMs are maps of effectiveness studies, and so the primary studies are only studies of effects, that is impact evaluations. Unlike systematic reviews, evidence maps may include on‐going studies. This is especially the case for on‐going reviews since registering review protocols is common practice, which remains less the case for primary studies. 3ie staff have argued that the term EGM should be restricted to this sort of map (Snilstveit, Vojtkova, Bhavsar, & Gaarder, 2013). compare Evidence Gap Maps, as supported by the International Initiative for Impact Evaluation (3ie), with other evidence mapping synthesis products, such as scoping reviews, systematic review and rapid evidence assessment. They propose that gap maps focus on studies assessing intervention effectiveness, as well as systematic reviews of such studies, while other mapping/ scoping approaches may include a broader range of evidence to address questions other than those of intervention effectiveness.

Epistemonikos uses systematic reviews as the fundamental piece of the database architecture and includes articles linked to an included systematic review i.e. overviews of reviews including evidence‐based policy briefs and guidelines, primary studies included in systematic reviews, and structured summaries of that evidence.

Some agencies restrict their included studies in specific ways. UNICEF‐Innocenti exclude transferable skills and youth employment‐related interventions and outcomes as other EGMs address these. Beam exchange evidence gap includes empirical data with studies not earlier than 2000 as this when original framework document for making markets work better for the poor (M4P) was developed.

### Critical appraisal of included studies

Critical appraisal for the quality of studies indicates the confidence in the study findings. For example, in 3ie maps systematic reviews are colour coded green, orange and red corresponding to high, medium and low confidence in study findings. 3ie does not conduct critical appraisal of primary studies on the grounds that it would be too time consuming given the time frame in which maps are usually produced (3‐6 months). In 2017, BEAM Exchange updated the original evidence inclusion protocol to include an additional evidence quality grading system, which assesses all evidence documents as either ‘high confidence’ (represented by purple ‘bubbles’) or ‘low confidence’ (represented by orange ‘bubbles’).

Critical appraisal is applied using a pre‐specific critical appraisal tool or chechklist. 3ie, UNICEF‐Innocenti and Sightsavers use and adapted version of the SURE checklist, and in SBU and ODI apply the AMSTAR checklist. Epistemonikos presents critical appraisal of articles included in structured summaries. For CEE, critical appraisal (the formalised assessment of reliability and risk of bias in individual studies) may be performed to some extent, but this is typically restricted to an assessment of study internal validity (quality or susceptibility to bias), since external validity (generalisability) cannot typically be assessed for the broad topics often investigated with systematic maps. The Evidence Based Policing Matrix reports primary studies only, for which critical appraisal is performed using Scientific Methods Scale (SMS). Critical appraisal is not presented by SCIE and GEMI.

Our recommendation that critical appraisal of all included studies is desirable, and should be performed unless resources do not allow it.

### Screening and search strategy

***Search*** is ***comprehensive and systematic***, similar to systematic review search strategy and is sometimes more ***purposive*** for Impact Evaluations.

The choice of ***source*** varies among organisations and may include:

- database of systematic reviews
- key evidence repositories
- academic database
- reference snowballing
- hand searching
- expert consultations
- websites of professional and government organisation.

There is considerable overlap in the ***search strategy*** adopted by these organizations for evidence maps and there is uniform adoption of a comprehensive and systematic search and involves search across all the sources found relevant for the research question. However in ‘3ie’ search for IE may be more purposive.

Key difference in the ***source*** for search is that, ‘3ie’ does not involve hand searching of journals or literatures and ‘Evidence Based Policing Matrix’ specifically involves searching website of professional and government organization.

In Epistemonikos, articles coming from different databases are de‐duplicated using software and a manual revision of borderline cases.

### Coding and data extraction

Coding captures key features of included studies allowing analysis of the map. The more data are captured then the more dimensions the map can have, or filters be applied to restrict the map to a subset of the evidence by user‐determined criteria.

The following domains have been used by different organisations for classifying included studies (Table 3 provides an overview of which organizations use which study characteristics):

**Table 3:** Information coded from included studies

	**Intervention category**	**Outcome**	**Status: Ongoing /completed**	**Inclusion criteria/PICOs**	**Study design**	**Critical appraisal**	**Direction, size and statistical significance of effects**
**3ie**Snilstveit, Vojtkova, Bhavsar, & Gaarder, 2013)	X	X	X	X	X		
**GEMI** (Bragge, et al., 2011)	X	X		X	X		
**CEE** (James, Haddaway, & Randall, 2016)	X	X		X	X	X	
**EPPI‐Centre** (Gough, Kiwan, Sutcliffe, Simpson, & Houghton, 2003)	X	X		X	X	X	
**SCIE** (Clapton, Rutter, & Sharif, 2009)	X	X		X	X	X	
**SBU** (Christel Hellberg)	X	X	X	X	X	X	
**EBPM** (Lum, Koper, & Telep, 2011)	X	X			X		X
**Department of Veteran Affairs** Hempel, et al., 2014)	X	X		X	X		
**Epistemonikos** (Rada, Pérez, & Capurro, 2012)	X	X		X	X		
**IZA World of Labour** (Giovanni, 2014)	X	X		X	X		
**Sightsaver** (Evidence Gap Maps)	X	X	X	X	X		
**Beam Exchange** (Evidence Map)	X	X			X	X	
**IRC** (Framing Organisational Effectiveness: The IRC's Approach to Outcomes and Evidence)	X	X		X	X		
**ODI** (Marcus & Cunningham, 2016)	X	X		X	X		
**UNICEF Innocenti** (Bakrania & Ghimire, 2017)	X	X		X	X

- Intervention categories
- outcome categories
- status of the study: completed or ongoing
- geographical coverage of the study, where applicable
- inclusion criteria (PICOs) of included systematic reviews
- primary study design (RCT, PSM etc.)
- critical appraisal of included studies
- information about intervention effectiveness

In addition, study publication details are of course coded so that users can locate the evidence underlying the map. How these data are represented is discussed in the next section on reporting / presentation.

Typically, ***Intervention categories, Outcome categories*** and ***study designs*** are used for coding in all the methodologies of evidence mapping. Representation of Outcome categories in 3ie gap maps and Evidence Based Policing Matrix differ, in Evidence Based Policing Matrix, key intervention dimensions (specificity of strategy, level of pro‐activity of intervention, nature of target), evaluation design and outcome (direction and statistical significance of effect are used for data extraction while in 3ie, studies are coded according to relevant intervention and outcome categories included in the framework. ***Detailed study characteristics*** are included in GEM maps and use codes from the International Classification of Functioning, Disability and Health (ICF). The EBPM is also an exception in being the only map to report what the evidence says (size, direction and significance of effects).

In Epistemonikos, once the document is uploaded, meta‐data and relational panel allows entering document metadata such as document's category like primary studies, systematic reviews, overviews and the document's relation.

### Presentation and reporting

Five approaches have been used for presentation of evidence maps by the different organisations: the 5D bubble plot, a 3D‐matrix, a 2D‐matrix, as geographical map and descriptive report with/without visualisation. In the latter two cases additional dimensions may be added through use of different colors, symbols and size to plot studies in the map.

Each of these approaches is described in turn.

#### 5D‐bubble plot format

The 5D‐Bubble format is used by Evidence Based Synthesis Program. The bubble plot shows five dimensions to display information: the x and y axes show the effect and size of the literature respectively. Each bubble reflects three dimensions: there is a separate bubble of each intervention, with the size of the bubble representing the number of reviews for that intervention and the color of the bubble the intervention category. Figure shows an example of bubble plot.

**Figure 2:**
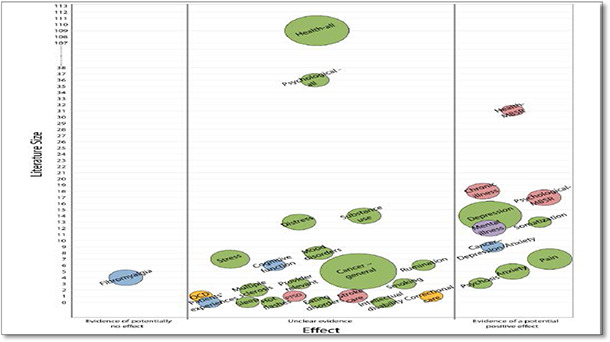
Evidence map on mindfulness by evidence based synthesis program Source: Evidence Based Synthesis Program (https://www.ncbi.nlm.nih.gov/pubmed/25577939)

### 3D‐matrix

A 3D‐Matrix is used by ‘Evidence Based Policing Matrix’ to display single studies in three dimensions: intervention categories on the X axis (defined by target: individual, group etc.), the specificity of the intervention from general to focused, and its degree of proactivity, from reactive to highly proactive. Each study is plotted by a circle or triangle, color coded to show study findings, i.e. effects are added as a fourth dimension in the map. This approach shows of studies which, if they are for a common intervention, are referred to as “realms of effectiveness.” These realms of effectiveness are said to provide insights into the nature and commonalities of effective police strategies and can be used by police agencies to guide various aspects of their operations. However, this approach is not a formal evidence synthesis, and encourages a vote‐counting approach to assess the evidence.

**Figure 3:**
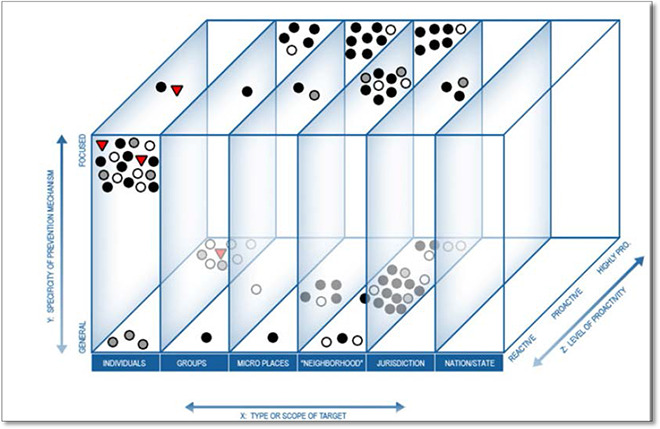
The matrix mapped with 97 police intervention studies Source: Evidence Based Policing Matrix (https://cebcp.org/evidence‐based‐policing/the‐matrix/)

### 2D‐Intervention‐outcome framework

#### 3ie evidence gap map

3ie present their evidence gap maps in a 2D‐intervention‐outcome framework that provides links to summaries of included studies. Traffic light color coding is used to indicate the quality of the systematic review evidence in the gap map so that users can visually assess the state of the evidence in the field use symbols. The same assessment is not made for primary studies. The size of each circle represents the number of studies.

**Figure 4:**
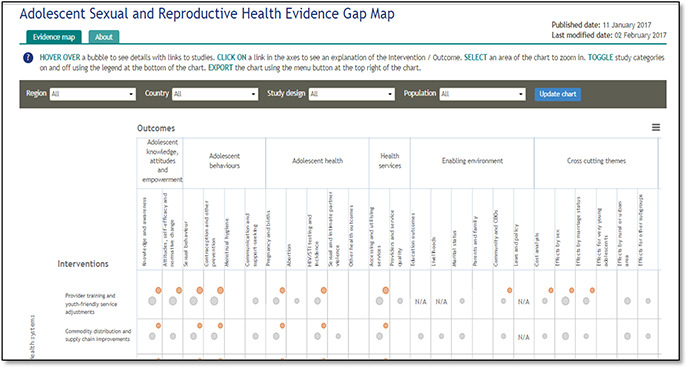
Adolescent and sexual health EGM Source: 3ie Adolescent, sexual and reproductive health evidence gap map: http://gapmaps.3ieimpact.org/evidence‐maps/adolescent‐sexual‐and‐reproductive‐health‐evidence‐gap‐map

#### UNICEF Innocenti

**Figure 5:**
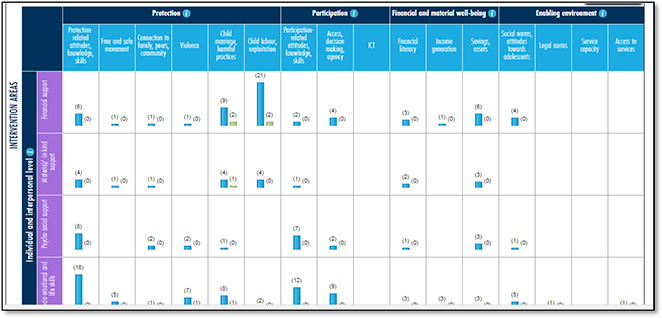
Evidence Gap Map on adolescent well‐being in low‐ and middle‐income countries: protection, participation, and financial and material well‐being Source: UNICEF Innocenti (https://www.unicef‐irc.org/evidence‐gap‐map/)

#### Sightsaver EGM

Sightsaver EGMs are based on a matrix framework designed to capture evidence on specific interventions or focus areas. The focus areas for which reviews were identified are found along the x axis, and the strength of evidence is shown along the y axis. The bubbles in the matrix cells denote the existence of a systematic or literature review examining the relevant focus area. Clicking on a bubble will open up a summary page. The strength of evidence presented in the reviews is categorised as:

- **strong** if the review found strong evidence in response to the research question or outcome
- **inconclusive** if the review reported mixed results
- **weak** if the review found weak or no evidence in response to the research question or outcome

**Figure 6:**
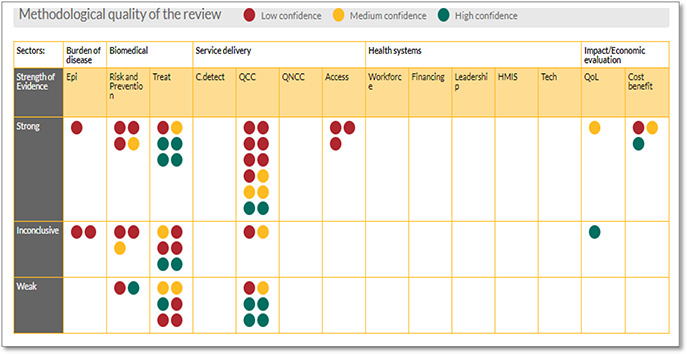
Cataract gap map Source: Sightsaver (https://research.sightsavers.net/gap‐maps/cataract‐gap‐map/)

#### Beam exchange EGM

The BEAM Evidence Map and database compiles and presents robust evidence on the impact and effectiveness of market system approaches.

The vertical axis: the type of intervention which contributed to the results described in the document.

Horizontal axis: the results level of the evidence which the document contains

**Figure 7:**
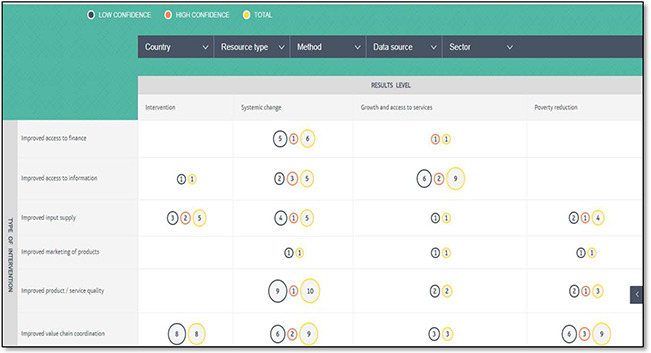
Beam Exchange evidence map Source: Beam Exchange (https://beamexchange.org/resources/evidence‐map/)

#### Matrix of evidence

A matrix of evidence is a table displaying all the systematic reviews answering a question, and all of the studies included in these reviews. The matrix displays the systematic reviews in the y‐axis, and the studies included in those reviews in the x‐axis.

All the systematic reviews in the table address the question of interest (i.e. Melatonin for jet lag) or a broader question that includes the question of interest (i.e. Melatonin for promoting a healthy sleep).

**Figure 8:**
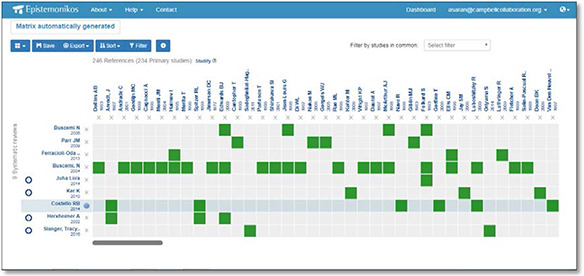
Matrix of Evidence (Epistemonikos) Source: Epistemonikos (https://www.epistemonikos.org/en/documents/934b7d3da7216f697504b80170b87b58ec04709b/matrix)

#### IRC evidence map

IRC evidence maps are not available as online tool but are available in downloadable Excel format.

**Figure 9:**
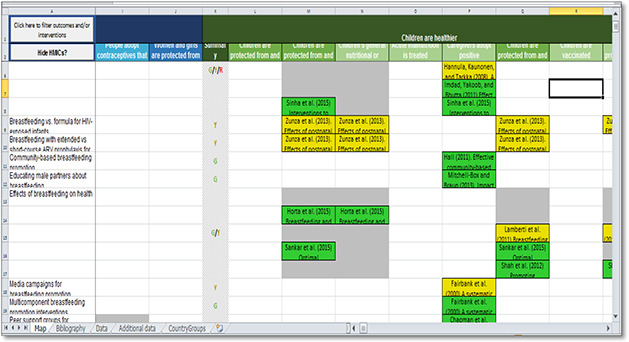
Health map Source: IRC (https://www.rescue.org/resource/strategy‐2020‐outcomes‐and‐evidence‐framework‐evidence‐maps)

The cells are color‐coded based on the study's conclusion:

- Green: Evidence of meaningful, positive impact in a given context
- Yellow: Not yet conclusive evidence of meaningful, positive impact in a given context
- Red: Evidence of no or negative impact in a given context
- Impact evaluations are put in as light yellow

#### Overseas Development Institute (ODI)

**Figure 10:**
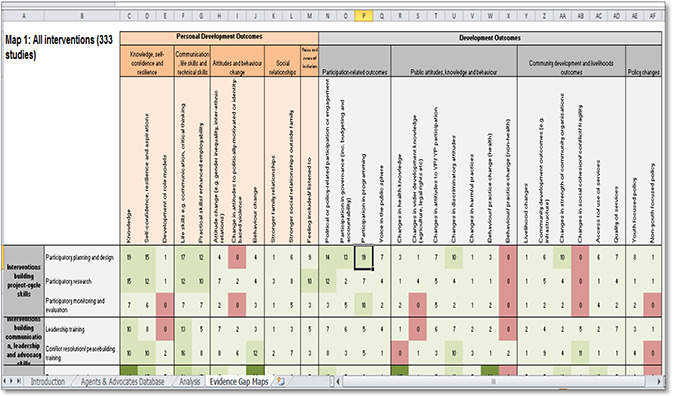
Young people as agents and advocates of development Source: ODI (https://www.odi.org/publications/10653‐young‐people‐agents‐and‐advocates‐development)

### Presenting the map as a geographical map

IZA World of Labor article map indicates for which countries empirical evidence on a specific topic exists. The list of key and additional references provided in the article provides the basis for this map.

If empirical evidence exists for one particular country, that country will be shown in a different color, varying from yellow to different shades of green. The colour of the country indicate how it is classified according to the classifications used in The Global Competitiveness Report (GCR) 2014–2015 (Schwab, 2014) by the World Economic Forum's (WEF) Center for Global Competitiveness and Performance. A country that is not categorized in the GCR will be colored gray. This feature can be seen as assisting users assist the transferability of findings given the study context, as captured by its GCR classification.

The number shown inside the pin indicates how many relevant academic studies address this policy question.

**Figure 11:**
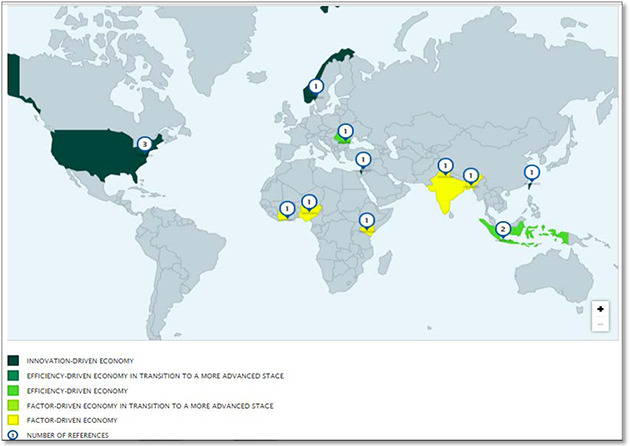
Do immigrant workers depress the wages of native workers? Evidence map Source: IZA (https://wol.iza.org/articles/do‐immigrant‐workers‐depress‐the‐wages‐of‐native‐workers/map)

An additional feature is that maps may be available online, with the possibility that the user can click on a map entry to access either a record giving study information, i.e. publication details and a summary of the study. These summaries typically include information on:

- Geographical location
- Background
- Methods
- Main findings
- size of the treatment effect
- the individual included clinical indications
- the assessed outcomes
- characteristics of the identified reviews
- the comparator against which the treatment effect was estimate

### Descriptive reports with/without visualisation

Some agencies do not provide visualization but present the results of the mapping in a descriptive report. A descriptive report describes the overall amount of evidence and its characteristics, such as geographical distribution and which study designs have been used. The reports identify knowledge gaps and knowledge clusters.

#### Descriptive report and tables

Agencies like SBU present in the form of a descriptive report and a table to show where there is evidence as shown in the figure below.

**Figure 12:**
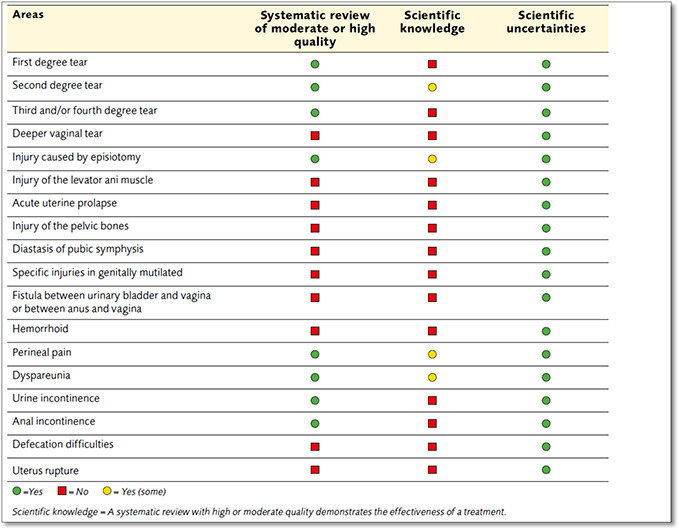
SBU scientific knowledge, a systematic review with high or moderate quality demonstrating the effectiveness of treatment Source: Treatment of maternal birth injuries following vaginal birth, SBU (https://www.sbu.se/en/publications/sbu‐kartlagger/treatment‐of‐maternal‐birth‐injuries‐following‐vaginal‐birth/)

#### Descriptive report and ‘output tables’

Global Evidence Mapping Initiative presents ‘output tables’ called maps along with descriptive report.

**Figure 13:**
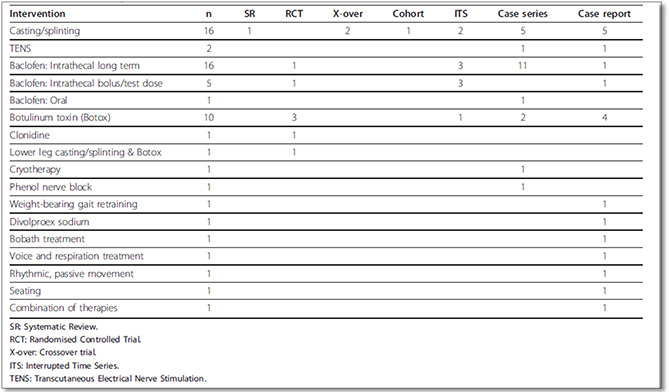
Interventions and study design’ output: Prevention and management of skeletal muscle spasticity in the rehabilitation phase of TBI Source: Global Evidence Mapping Initiative (https://www.ncbi.nlm.nih.gov/pmc/articles/PMC3141802/)

#### Descriptive report with Pivot tables and charts

Collaboration for Environmental Evidence use pivot tables and pivot charts to easily visualize the quantity (and quality if assessed) of evidence across a suite of meta‐data variables. Sometimes they present study meta‐data as a layer within a geographical information system (GIS). That may be a simple world map showing the location and number of included studies .

Descriptive reports outlining methodologies are used by ‘Global Evidence Mapping Initiative’, ‘Social Care Institute for Excellence’, ‘Collaboration for Environmental Evidence’ and ‘Evidence for Policy and Practice Information and Coordinating Centre (EPPI‐Centre)’. Some organizations, such as 3ie and the Veterans' Association, which provide visualizations, also provide a descriptive report.

### Link to searchable bibliographic database

There are variations in whether the evidence can be accessed through the map. The interactive nature of 3ie maps, for example, means the user can access a database record for each included study, which in turn has a link to the study itself, though that may be behind a paywall. Link to summary of findings and conclusion is provided with IRC, Beam Exchange, ODI and Sightsavers.

### Use

Evidence map have varied use, depending on the mandate of organizations:

i. **Research:** To describe the nature, characteristics and volume of research in a particular area and inform research design. Also to set agenda for future research.
ii. **Systematic review planning:** initial steps in the planning of a systematic review
iii. **Identify evidence gaps:** To identify evidence gaps by comparing the key research questions identified by stakeholders with the available literature.
iv. **Funding:** used by funders for evaluating the need for further funded research when reviewing a grant application
v. **Policy decisions:** To inform policy and practice guide the formulation and selection of strategies in policing, develop an agenda for future policing research and serve as a practice‐oriented research translation tool that may better facilitate the adoption of evidence‐based policing and evidence‐based funding.

Evidence maps are used to ***inform research*** for commissioning and facilitating the use of evidence to inform decision making by ‘3ie’, ‘The Global Evidence Mapping Initiative’, ‘Social Care Institute for Excellence’, ‘Collaboration for Environmental Evidence’, ‘Evidence based Synthesis Program’ and ‘Evidence Based Policing Matrix’..

‘Evidence for Policy and Practice Information and Co‐ordinating Centre’, use evidence map as a part of the initial steps in ***systematic review planning***, or even to help make a decision about whether or not to undertake a systematic review.

Evidence maps have also been used to ***identify evidence gaps*** by ‘3ie’, Social Care Institute for Excellence’, ‘Collaboration for Environmental Evidence, ‘Evidence based Synthesis Program’. For example, SBU, used findings as to gaps for evidence for treating ADHD as the basis for a consultation exercise to identify the top ten research priorities (Jacobson et al. 2016).

Have been used for annual grant programme and ***funding*** decisions by ‘Swedish Agency For Health Technology Assessment and Assessment of Social Services’, The Global Evidence Mapping Initiative’ ‘Collaboration for Environmental Evidence’ and ‘3ie’.

‘3ie’ evidence map and ‘Evidence Based Policing Matrix’ are used to inform ***policy decision*** and as translation tool for adoption of evidence based research and funding, particularly due to visual representation of evidence and gaps.

**Table 4:** Intended use of evidence maps

Organisation	Inform research	Initial step in systematic review planning	Identifying evidence gaps	Funding decisions	Inform policy decisions	Identify ‘what works’
3ie	X		X	X	X	
GEMI	X			X	X	
CEE	X	X	X	X	X	
EPPI‐Centre		X			X	
SCIE	X		X			
SBU	X	X	X			
EBPM	X			X	X	X
Department of Veteran Affairs	X		X		X	
Epistemonikos	X			X	X	
IZA World of labour map	X				X	
Sight saver	X		X		X	
Beam exchange	X		X	X	X	
IRC	X					
ODI	X				X	
UNICEF Innocenti	X		X	X	X	

### Updating or maintenance

The search for an evidence map is undertaken up to a particular point of time. The map thus becomes out of date as new evidence accumulates. Maps more than two to three years old are less useful as a guide to research priorities, though this is partly mitigated if the map has successfully captured on‐going studies. Hence, as with systematic reviews, to stay relevant maps need to be updated or maintained.

Of the agencies reviewed, the Evidence Based Policing Matrix and Epistemonikos are updated on a regular basis. SBU updates the evidence gap in their database but not the evidence maps.

Some like Global Evidence Mapping Initiative, Collaboration for Environmental Evidence has a policy that maps should be updated, but there is no mechanism for ensuring that this happens.

### Comparison of evidence maps and systematic reviews

Based on the above discussion Table 5 summarizes the similarities and differences between evidence and gap maps and systematic reviews. There are many points of similarity: evidence and gap maps are systematic, so need an ex ante protocol with an explicit search strategy with clear inclusion and exclusion criteria. The main difference is the more limited coding of studies carried out for a map, since it only reports what studies there are not what it says.

**Table 5:** Comparison of systematic reviews and evidence and gap maps

	**Systematic review**	**Evidence and gap map**	**Comparison**
Question setting	Often restricted to a single intervention, and a limited range of outcomes. A PICOS is specified to guide study inclusion criteria.	Broad scope of interventions across a sector or sub‐sector, with full range of outcomes across causal chain. A PICOS is specified to guide study inclusion criteria.	EGMs are broader in scope than systematic reviews.
Search strategy	A comprehensive and systematic search for primary studies meeting the inclusion criteria (and not exclusion criteria)	A comprehensive and systematic search for systematic reviews and primary studies meeting the inclusion criteria (and not exclusion criteria)	No difference in approach. EGMs search for systematic reviews as well as primary studies.
Screening	Identified studies screened against inclusion and exclusion criteria	Identified studies screened against inclusion and exclusion criteria	No difference in approach
Coding and data extraction	Coding of study and intervention characteristics, moderators and data extraction of effect sized and related statistics	Coding of a limited number of study and intervention characteristics	EGMs require coding of less data than systematic reviews
Critical appraisal	Assessment of quality of included studies using a critical appraisal instrument	Critical appraisal may not be done, but is recommended	Critical appraisal is optional for EGMs
Evidence synthesis	Statistical or narrative synthesis of the evidence	Not done	EGMs do not synthesize the evidence
Reporting	Systematic reporting of evidence	Graphical representation of map availability of evidence. Descriptive overview of map.	Systematic reviews summarize what the evidence says. EGMs only summarize what evidence is available.
Use	To inform policy and practice	To inform research priorities and research funding	Systematic reviews are to inform policy, and EGMS primarily to inform research priorities.

### General principles for evidence maps

Based on the discussion we propose the following principles for producing evidence maps:

- Clearly determine the intended purpose of the planned evidence map, including the type of evidence to be included (e.g. effectiveness) and the planned structure of the map
- Define the scope of the map, which should be stated as a clear title
- Have an ex ante search strategy and coding form (which should include stakeholder engagement in defining the map framework and then be piloted)
- Include on‐going studies in the map by searching registries for primary studies and reviews
- Include critical appraisal of the quality of evidence
- Have a visual representation in at least two dimensions, with possible additional dimensions or filters
- Be accompanied by a descriptive report.

Be updated within three years of last completion, preferably annually if scope is broad or field changing rapidly.

## ANNEXES

**Evidence and gap map definitions**

**Table:** 

**Organisation**	**EGM definitions**
EBPM	The Evidence‐Based Policing Matrix is a research‐to‐practice translation tool that organizes moderate to very rigorous evaluations of police interventions visually, allowing agencies and researchers to view the field of research in this area.(Lum, Koper, & Telep, 2011)
EPPI	A systematic map is defined as ‘a classification and description that aims primarily to illustrate the kinds of studies that exist’ (EPPI‐Centre, 2007) (Dickson, Vigurs, & Newman, 2013)
SCIE	Systematic mapping is a transparent technique for describing research literature on a broad topic. A map provides both a searchable database for researchers and policy makers, and a stand‐alone piece of work describing the literature on that topic. (Clapton, Rutter, & Sharif, 2009)
3IE	3IE EGMs are collections of evidence on the effects of development policies and programmes in a particular sector, sub‐sector or thematic area, structured around a framework of interventions and outcomes. They provide a graphical display of existing and ongoing systematic reviews and impact evaluations.(Snilstveit, Vojtkova, Bhavsar, & Gaarder, 2013)
GEMI	Evidence mapping: The systematic organisation and illustration of a broad field of research evidence. (Bragge, et al., 2011) (Arksey & O'Malley, 2005)
CEE	Systematic mapping is a robust, repeatable and transparent scientific method used to identify, categorise and map available literature relevant to a topic.(James, Haddaway, & Randall, 2016)
SBU	Per the definition used at SBU, a scientific evidence gap exists when one or several systematic reviews of good quality, show an unsure effect of a method or an intervention or when no systematic review of good quality evaluating the method or intervention is to be found.
Department of veteran affairs	The evidence map—a visual overview of a systematic review of systematic reviews—is a new and unique review product that shows graphically, at a glance, the volume and focus of a research area through bubble color, size, and location. (Solloway, et al., 2016)
Epistemonikos	Epistemonikos project is a free, relational, collaborative, multilingual database of health evidence, established six years ago with the aim of gathering all what is usually considered health evidence useful for decision‐making (systematic reviews, studies, overviews, guidelines, structured summaries, etc), and to organise it by health question (also by specialty and subspecialty among other options). The project combines cutting edge software with the human effort of more than 500 collaborators, and its growth has exceeded even the most optimistic expectations. (Rada, Pérez, & Capurro, 2012)
IZA world of labour	Each IZA World of Labor article has a map that indicates for which countries empirical evidence on a specific topic exists. The list of key and additional references provided in the article provides the basis for this map. If empirical evidence exists for one particular country, that country will be shown in a different color, varying from yellow to different shades of green. The color used indicates the country's development status based on the country classification The number shown inside the pin indicates how many relevant academic studies address this policy question.
IRC	IRC's Evidence Maps, an adaptation of the Gap Map approach developed by the International Initiative for Impact Evaluation (3ie). Evidence Maps organize and visually present the best available research evidence at every step of the theory of change to best determine what interventions do and do not work in each outcome area. EGM is simply a grid, with outcomes of interest defining the columns (e.g. literacy, numeracy, attendance) and interventions of interest defining the rows (e.g. cash transfers, early childhood nutrition, teacher training).
Beam exchange	This map consolidates resources that describe the impact and effectiveness of programmes that use a market systems approach. All resources in the map meet the primary inclusion criteria. ‘Low confidence’ resources partially meet the secondary criteria. ‘High confidence’ resources fully meet all the criteria. Hover over a bubble to see the resources, or use the filters to narrow your search.
Sightsavers	Evidence gap maps (EGMs) are a visual tool for presenting the state of evidence relevant to international development in particular thematic areas. EGMs summarise, appraise, and present evidence from systematic or literature reviews. The aim is to make the available evidence accessible in a user‐friendly format and to highlight gaps for future research. For each review the maps display its summary, the strength of evidence and the methodological quality of the review.
ODI	This evidence map brings together the available evidence examining the effectiveness of interventions that support young people as either ‘advocates’ or ‘agents’ of development
UNICEF Innocenti	An EGM intends to inform policy and practice by offering a snapshot of evidence on a topic or theme at a given point in time. Using a matrix of intervention types and outcomes, an EGM maps empirical evidence to show where it is strong and where the gaps lie.

## Footnotes

1 The exclusion of a component for an organization does not mean its maps do not have that feature, but it is not included in their published definition.
